# Cancer of the Uterus

**Published:** 1888-06

**Authors:** 


### Reviews of Books.

*Cancer of the Uterus.* By JOHN WILLIAMS, M.D., F.R.C.P. Pp. 119. London: H. K. LEWIS. 1888.

THE pathological parts of this work, and particularly the admirable plates, form a useful, if not brilliant, contribution to our knowledge of the natural history of cancer of the uterus. Although it cannot be said that the author has been able to draw any conclusions that are either novel or beneficial from this part of his work, yet he deserves credit for having put on record a series of careful clinical and pathological studies which others following him, with more ambitious aims, may find useful.

From the practical point of view the work is not commendable. It is essentially a special plea for extirpation of the cervix, as against extirpation of the whole uterus; and has many of the usual bad features of special pleading. Examples could be shown where he is wrong as to facts; where he is partial and one-sided as to his selection of statistics; and where he is erratic or biassed in his arguments. We know a good deal of cancer of the breast. What should we say of the capacity for fair argument of a man who said that because the mortality of making a clean sweep of the whole organ with the overlying skin was greater than after the excision of one affected lobe, therefore the operation for all cancers of the breast should be excision of only the palpably diseased part? Is no account whatever to be taken of the extent of the disease? nor of the unity for disease as for function of the whole gland? Is the percentage of recurrence to be measured by the lines of an incision based on surgical or anatomical convenience, rather than by the extent of the invading disease? No reasonable man will believe that, in a given case of cancer of the uterus, recurrence is more likely to follow total removal than partial removal. These are not the lines to argue from: we must base our reasoning and our practice on the limits of the disease and on sound logic. Where removal of the cervix gives a fair chance of success, immediate and remote, let us remove the cervix; where the opposite condition holds and the operation is proper at all, let us remove the whole uterus. If, in the great majority of cases, the minor operation is preferable, this is no reason why the major operation should be utterly condemned.

